# The effect of probiotic supplementation combined with aerobic exercise on the antioxidant capacity of college students

**DOI:** 10.3389/fphys.2025.1586888

**Published:** 2025-06-02

**Authors:** Tong Wu, Yingfeng Chen, Kai Zhao, Chenzhe Liu, Wei Jiang

**Affiliations:** ^1^ Department of Exercise Physiology, School of Sport Science, Beijing Sport University, Beijing, China; ^2^ Chongqing Bishan Bashu Middle School, Chongqing, China; ^3^ China Volleyball Sport College, Beijing Sport University, Beijing, China; ^4^ Laboratory of Sports Stress and Adaptation of General Administration of Sport, Beijing, China

**Keywords:** probiotics, aerobic exercise, oxidative stress, antioxidant capacity, high-intensity interval training

## Abstract

**Introduction:**

This study examined the effects of probiotic supplementation alone or combined with aerobic exercise on antioxidant capacity and oxidative stress after high-intensity interval exercise (HIIE) in college students.

**Methods:**

Thirty male college students were divided into three groups: control (C), probiotic (P), and combined probiotic and exercise (PE). The 6-week intervention involved moderate-intensity cycling three times a week. All participants underwent a single session of HIIE protocol. The tests for maximal oxygen uptake (VO_2_max), elimination rate of lactic acid (ER), blood oxidative stress markers, and blood rheology were performed.

**Results:**

A decrease in superoxide dismutase (SOD) activity was observed at baseline in the P and PE groups (*P* < 0.01), while significantly increased glutathione peroxidase (GSH-Px) activity and reduced catalase activity were found in the PE group (*P* < 0.05). In the P and PE groups, SOD activity (*P* < 0.01) and total antioxidant capacity (T-AOC) level (*P* < 0.01) were significantly elevated after HIIE. The T-AOC level significantly increased from 0.47 ± 0.03 umol Trolox/mL to 0.78 ± 0.07 umol Trolox/mL in the P group and from 0.56 ± 0.04 umol Trolox/mL to 0.82 ± 0.05 umol Trolox/mL in the PE group. The 8-OHdG level increased significantly in both the C and P groups (*P* < 0.05), but remained unchanged in the PE group after the intervention. High shear rate whole blood viscosity was significantly decreased in the P and PE groups (*P* < 0.05). Additionally, a notable decline in plasma viscosity was observed in the PE group. After the intervention, medium and high shear rate whole blood viscosity levels (*P* < 0.05) were significantly lower in the PE group than in the C group, and plasma viscosity was dropped by 28.64% (*P* < 0.05). Following the intervention, a significant elevation in VO_2_max was only observed in the PE group from 38.14 ± 3.11 to 44.5 ± 2.94 mL/kg/min (*P* < 0.05), with a subsequent increase in ER detected after HIIE (*P* < 0.05).

**Discussion:**

These findings indicate that combining probiotics with aerobic exercise enhances antioxidant and aerobic capacity more effectively than probiotics alone.

## Introduction

Normally, the generation and clearance of reactive oxygen species within the human system are in dynamic equilibrium, which plays a positive role in sustaining normal body functions. Exercise serves as an important stressor of oxidative stress and plays a dual role in regulating the body’s redox system. After high-intensity strenuous exercise, especially high-intensity interval exercise (HIIE), The body’s overproduction of reactive oxygen species (ROS) induces a rise in oxidative stress (Sousa et al., 2025). Oxidative stress can lead to muscle damage, exercise-induced fatigue, and impaired athletic performance (Martinez-Canton et al., 2024). In young populations, oxidative stress induced by a single bout of high-intensity exercise (e.g., an acute session of HIIE) may impair recovery and hinder sustained exercise performance. (Cipryan, 2018; Lomiwes et al., 2024). In contrast, long-term HIIE has been shown to induce beneficial adaptations, including enhanced antioxidant enzyme activity and reduced oxidative stress levels, contributing to improved resilience and exercise capacity over time (Guo et al., 2025; Sarvasti et al., 2020). Consequently, numerous researchers have been exploring appropriate interventions to mitigate the level of oxidative stress and improve the body’s antioxidant capacity after high-intensity exercise (Souglis et al., 2023; Li et al., 2025; Wang et al., 2025).

When taken in adequate amounts, probiotics—living microorganisms—can positively influence overall health (Sarita et al., 2024). Common strains include *Lactobacillus* bulgaricus, *Lactobacillus* acidophilus, Bifidobacterium lactis, and *Lactobacillus* casei. Pro-biotics are proven to confer health benefits to the host, such as alleviating gastrointestinal discomfort and the duration/severity of upper respiratory tract infections (Bertuccioli et al., 2024; Damianos et al., 2025), enhancing the intestinal mucosal barrier defense (Li et al., 2025), and improving immune function (Aykut et al., 2024; Mafe et al., 2025). Studies in recent years have shown that probiotic supplementation contributes to improving antioxidant capacity and reduces serum malondialdehyde (MDA)level in individuals with regular physical exercise (Sánchez Macarro et al., 2021; Musazadeh et al., 2024), triathletes (Huang et al., 2019), and cyclists (Michalickova et al., 2018). However, these studies cannot exclude the effects of the exercise, studies on untrained individuals are limited.

In addition to the intake of probiotics, endurance training has been shown to enhance the antioxidant system. In older women, 8 weeks of aerobic exercise led to a notable decrease in 8-hydroxy-2′-deoxyguanosine (8-OHdG), a DNA oxidative damage biomarker, while boosting total antioxidant capacity (T-AOC) (Zhao et al., 2023). Eight weeks of aerobic exercise significantly reduces levels of 8-OHdG, a biomarker of DNA oxidative damage, and increases T-AOC level in older women (Zarrindast et al., 2021). Therefore, combining probiotic supplementation with aerobic exercise may have synergistic effects in enhancing antioxidant capacity and alleviating oxidative stress associated with high-intensity sessions. However, differences in the specific effects of probiotic supplementation alone or probiotics combined with aerobic exercise in these areas are not known. In addition, probiotic supplementation has been reported to improve aerobic capacity (Imanian et al., 2024). Our previous study also found that probiotic supplementation improved lactate metabolism after exhaustive exercise in college football players (Zhang et al., 2023). Therefore, this study focuses on the effects of probiotic supplementation alone or in combination with aerobic exercise on antioxidant capacity, oxidative stress induced by HIIE, and aerobic capacity. This study’s outcomes are projected to furnish experimental backing for the role of probiotics in alleviating oxidative stress induced by physical activity.

### Study design and procedures

#### Participants

30 male undergraduate participants were selected from Beijing Sport University for this study. None of the participants had previous training experience. Given that aerobic exercise has been proven to significantly enhance antioxidant capacity (Zhao et al., 2023), this study did not establish a separate aerobic exercise group but instead focused on verifying the effects of probiotic supplementation alone or in combination with aerobic exercise. The subjects were randomly assigned to a placebo group (C), a probiotic group (P), and a probiotic combined with an aerobic exercise group (PE), ten persons per group. Throughout the experimental period, the participants continued with their usual diet.

### Probiotic administration

Probiotic supplements were provided by Beijing Scitop Bio-tech Shareholding Co. Ltd. (Beijing, China). The probiotics, delivering 2 g per packet, came as a powder. The C group received a placebo consisting of maltodextrin, while the P group was given daily probiotic supplements containing three probiotic species: *Lac-tobacillus casei Zhang* (≥8 × 10^9^CFU), *Bifidobacterium lactis V9* (≥6 × 10^9^CFU), and *Lactoba-cillus plantarum P-8* (≥6 × 10^9^CFU). The probiotic was consumed in cold water half an hour after lunch each day. During the experiment, the intake of other fermented foods was restricted, with a 2-h gap observed between taking antibiotics and supplements. Apart from consuming the same probiotics as the P group, the PE group participated in aerobic ex-ercise three times a week.

### Study protocol

**FIGURE 1:**
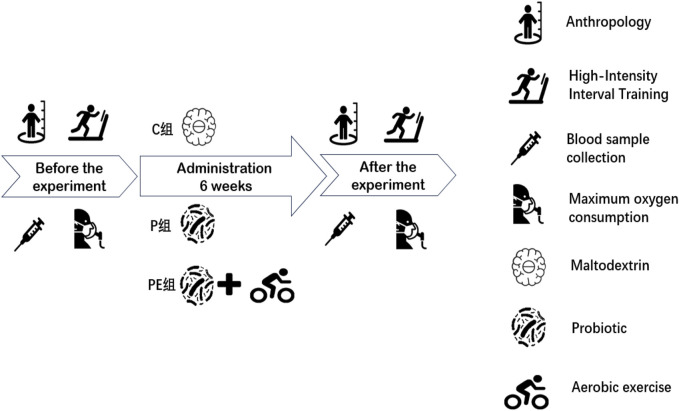
Experimental design.

The primary objective of this experiment was to examine the impact of probiotic supplementation on antioxidant capacity and oxidative stress in college students post high-intensity intermittent exercise, and to assess if a combined effect with aerobic exercise exists. The qualifications for participant inclusion were as follows: (1). No consumption of microbial supplements, probiotics, synbiotics, or foods with fermentation (like yogurt). (2). No training experience. The exclusion criteria included sensitivity to probiotic components, use of probiotics, and antibiotics within 1 month before the start of the study. Prior to the experiment, all participants filled out an informed consent form, volunteered for the study, and confirmed they could undergo all procedures. The experimental protocol was approved by the Ethics Committee of Beijing Sport University (approval number: no.2022215H). There were no significant differences in basic information between the C, P, and PE sub-groups concerning height, weight, age, body fat, body mass index (BMI), or maximal oxygen consumption (VO_2_max). (Table 1). The assessment duration lasted 6 weeks, and the methodology remained unchanged for both the baseline and final tests (Figure 1). All 30 participants participated in the assessments and data gathering.

**TABLE 1:** Research participant information.

Variable	C (n = 10)	P (n = 10)	PE (n = 10)	*P*-Value
Height (cm)	179.57 ± 2.37	176.71 ± 5.82	177.57 ± 6.0	0.598
Weight (kg)	78.69 ± 14.06	79.76 ± 11.30	81.96 ± 14.2	0.895
BMI (kg/m^2^)	24.78 ± 4.02	26.13 ± 3.52	25.31 ± 3.72	0.797
VO_2_max (mL/kg/min)	45.01 ± 3.9	40.86 ± 9.35	38.14 ± 8.23	0.255
Age (years)	22.29 ± 1.89	22.14 ± 1.95	21.71 ± 1.50	0.826

Results are expressed as the mean ± SEM. BMI, body mass index; VO_2_max, maximal oxygen uptake.

### Anthropometry

The height of the participants was measured using a standardized stadiometer, and their fasting body weight was recorded in the morning with a GMCS RCS IV portable scale.

#### High-intensity interval exercise test

Two days before the high-intensity interval exercise experiment, participants first underwent an exercise test to determine their maximum treadmill speed (MTV). The participants warmed up by walking on the treadmill at a speed of 6 km/h for 5 min. Subsequently, an incremental test was conducted with the treadmill set at a constant 1% incline, running at 7 km/h for 1 min, followed by a fixed increment of 1 km/h per minute until exhaustion. The high-intensity interval exercise protocol consisted of a 5-min warm-up at 50% MTV on the treadmill, followed by eight sets of high-intensity interval exercise, each set comprising 1 min of running at 100% MTV, immediately followed by a 1-min rest interval, during which the participants recovered by walking at 30% MTV (Dantas, Farias Junior et al., 2017), running for 1 min, and resting for 1 min. This protocol was designed as a single-session high-intensity interval exercise test to evaluate acute exercise-induced oxidative stress responses.

#### Aerobic exercise protocol

The PE group performed 30 min of moderate-intensity aerobic exercise three times a week using a stationary bicycle, with all sessions conducted between 2:00 p.m. and 4:00 p.m. The workload was set at 60%–69% of the individual’s maximum heart rate, aiming for a heart rate range of 120–140 beats per minute. While participants were pedaling the stationary bicycle, their heart rate was assessed with a heart rate monitoring device. The timing of exercise session started when the participant’s heart rate reached the lower limit of the moderate-intensity aerobic exercise range.

### Blood sampling and assay methods

Venous blood samples were collected from the anterior elbow vein of each participant at baseline and immediately after the high-intensity interval exercise test using standard venipuncture techniques. The blood was then centrifuged at high speed, and the plasma was stored at −80°C until further analysis. The activities of SOD, catalase (CAT), and glutathione peroxidase (GSH-Px), along with the level of 8-OHDG, were quantified using ELISA kits (Shanghai Jianglai Biotechnology, China). T-AOC was determined using a microplate meth-od (spectrophotometry).

### Maximal oxygen uptake

The VO_2_max test followed the standard Bruce protocol, in which participants under-went an incremental treadmill exercise test. Prior to testing, they were fitted with a compact respiratory gas collection mask and a Polar V800 heart rate (HR) monitor belt. After completing the warm-up, the test commenced according to the study protocol (Vilcant and Zeltser, 2024). Participants engaged in continuous exercise, and the COSMED software was used to calculate real-time speed, gradient, and gas metabolism. The test was stopped when the participant became exhausted and could no longer continue. The operator assessed the participant’s Rating of Perceived Exertion (RPE) at each stage of the test and continuously tracked their heart rate to ensure the incremental exercise was completed safely.

To confirm the achievement of VO_2_max, a minimum of three criteria from the list had to be satisfied: (1) A plateau in oxygen uptake or a decline in oxygen consumption as intensity increases; (2) no increase in heart rate with increasing intensity; (3) a respiratory exchange ratio reaching or approaching 1.15; (4) the RPE scale indicating a level of fatigue where the participant could no longer sustain the current workload.

### Lactic acid elimination rate

Following the high-intensity interval exercise test, samples were drawn from the par-ticipants’ fingertips at 0, 3, 5, 7, and 9 min post-exercise. The blood samples were analyzed using a portable blood lactate analyzer (Biosen S_line Lab, EKF Diagnostics Holdings Ltd., Germany). During blood collection, participants remained seated and were instructed to avoid activities like slow walking or stretching that could facilitate recovery. The lactic acid elimination rate (ER) was then estimated from the equation (Wang et al., 2019):

$$\text{ER}=\left(\text{ Lmax }‐\;L9\right)/\left(t9\;‐\text{ tmax}\right)$$

Here, ER represents the blood lactate elimination rate (mmol/Lmin); Lmax is the maximum blood lactate concentration after exercise (mmol/L); L9 is the blood lactate concentration at 9 min post-exercise (mmol/L); t9 is 9 min post-exercise (min); tmax is the time corresponding to the maximum blood lactate concentration (min).

### Blood rheology

Venous blood samples were collected from the participants at baseline, and blood rheology tests were conducted using a fully automatic rheometer (LBY-N6C, China).

### Statistical analysis

SPSS 25 software was employed to perform the analysis. Data are expressed as mean ± standard error (SEM). A one-way analysis of variance (ANOVA) was employed to compare the inter-group differences post-intervention and the rates of change. Paired sample t-tests were used to assess the changes within each group from pre-to post-intervention. Additionally, a two-way analysis of variance (ANOVA) was performed to examine the interaction effects between group and time. A statistical significance level of *P* < 0.05 was adopted.

## Results

### Effects of probiotics supplementation alone or in combination with aerobic exercise on oxidative stress

#### Effects on basal 8-OHdG level

**FIGURE 2:**
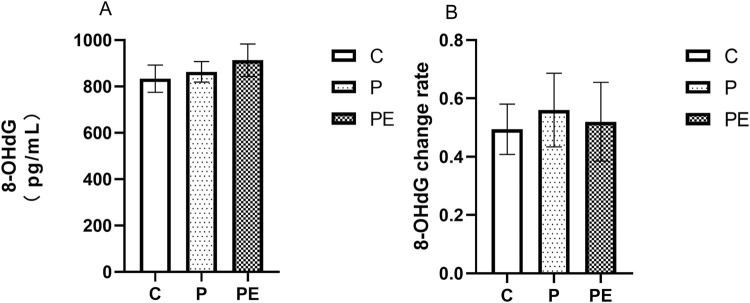
The effect of probiotics supplementation alone or in combination with aerobic exercise on 8-OHDG level at baseline in healthy college students. Results are reported as the mean ± SEM. C, control group (n = 10); P: probiotic group (n = 10); PE: probiotic and aerobic exercise group (n = 10) **(A)** 8-OHDG **(B)** 8-OHDG change rate. *: *P* < 0.05; **: *P* < 0.01.

This study assessed the impact of probiotic supplementation, either alone or combined with aerobic exercise, on 8-OHdG levels (an oxidative damage marker) at baseline. As shown in Figure 2A, 6 weeks of intervention resulted in no notable difference in 8-OHdG levels between the three groups (*P* = 0.63). Compared with pre-intervention levels, the rate of change in 8-OHdG levels showed no significant difference between groups post-intervention (*P* = 0.93, Figure 2B).

### Impact on the 8-OhdG level immediately after high-intense interval training

**FIGURE 3:**
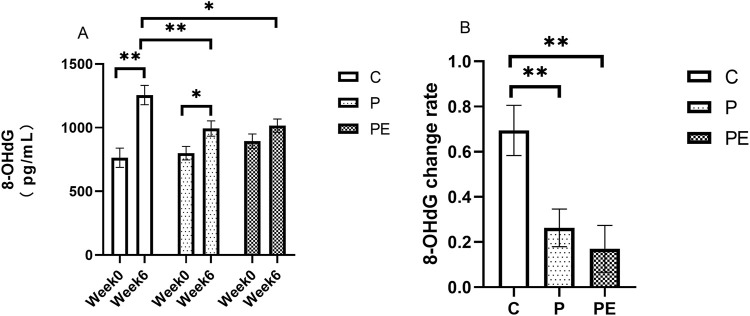
The effect of probiotics supplementation alone or in combination with aerobic exercise on 8-OHDG level immediately after high-intensity interval exercise in healthy college students. Results are reported as the mean ± SEM. C, control group (n = 10); P: probiotic group (n = 10); PE: probiotic and aerobic exercise group (n = 10) **(A)** 8-OHDG **(B)** 8-OHDG change rate. *: *P* < 0.05; **:*P* < 0.01.

Immediately after high-intensity interval exercise, the level of 8-OHdG was assessed in this study, both prior to the intervention and following 6 weeks of intervention. As shown in Figure 3A, the level of 8-OHdG increased significantly in the C and P groups after the intervention but remained unchanged in the PE group. The 8-OHdG level in the C group and P group was raised from 764.14 ± 75.69 pg/mL to 1,255.85 ± 75.75 pg/mL (*P* < 0.01) and from 799.77 ± 53.46 pg/mL to 992.93 ± 60.08 pg/mL (*P* < 0.05), respectively. As shown in Figure 3A, A significant increase in the 8-OHdG level was observed in the C group compared to both the P group and the PE group after the intervention. Additionally, as shown in Figure 3B, a significantly lower rate of change in 8-OHdG level was observed in the P and PE groups compared to the C group.

### Effects of probiotics supplementation alone or in combination with aerobic exercise on antioxidant capacity

#### Effects on antioxidant capacity at baseline

**FIGURE 4:**
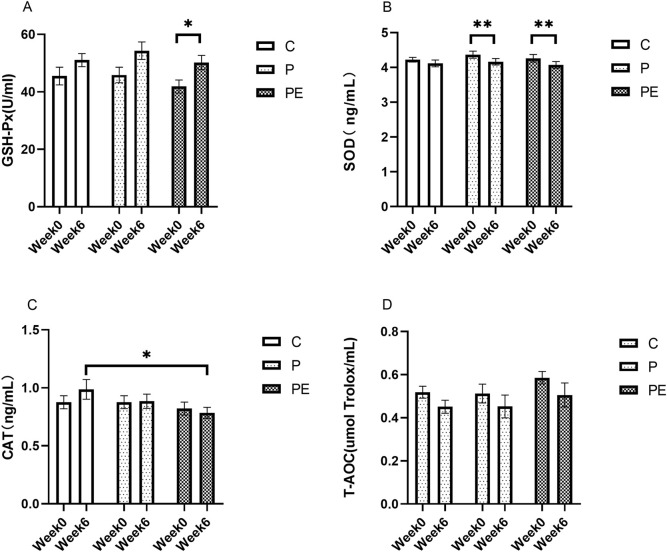
The impact of probiotics supplementation alone or in combination with aerobic exercise on antioxidant capacity at baseline in healthy college students. Results are reported as the mean ± SEM. C, control group (n = 10); P: probiotic group (n = 10); PE: probiotic and aerobic exercise group (n = 10). **(A)** GSH-Px. **(B)** SOD. **(C)** CAT. **(D)** T-AOC. *: *P* < 0.05; **: *P* < 0.01.

**FIGURE 5:**
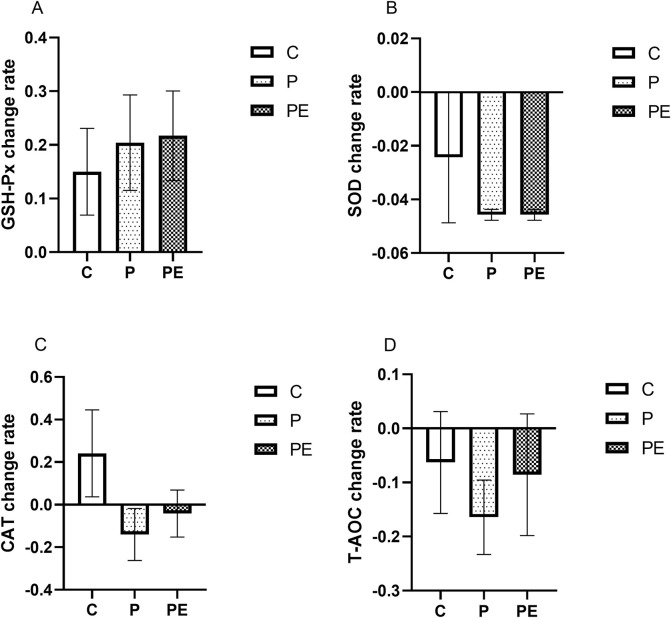
The impact of probiotics supplementation alone or in combination with exercise on the rate of change of antioxidant capacity at baseline in healthy college students. Results are reported as the mean ± SEM. C, control group (n = 10); P: probiotic group (n = 10); PE: probiotic and aerobic exercise group (n = 10). **(A)** GSH-Px change rate. **(B)** SOD change rate **(C)** CAT change rate. **(D)** T-AOC change rate. *:*P* < 0.05; **: *P* < 0.01.

An assessment was made of the effect of 6 weeks of probiotic supplementation, either alone or combined with aerobic exercise, on antioxidant indicators at baseline. As shown in Figure 4A, the GSH-Px activity was increased in the PE group from 41.86 ± 2.27 to 50.21 ± 2.48 U/mL when compared to pre-intervention levels (*P* < 0.05). However, no substantial differences were found in the GSH-Px activity between the P and C groups. Additionally, the SOD activity was decreased from 4.37 ± 0.10 to 4.16 ± 0.92 ng/mL in the P group (*P* < 0.01), and was reduced by 4.46% in the PE group (*P* < 0.01, Figure 4B), while was no significant changed in the C group. Moreover, no significant changes in the activity of CAT and T-AOC level were observed among all groups before and after the intervention (*P* > 0.05, Figures 4C, D). Furthermore, the rates of change in the activities of GSH-Px, SOD, CAT, and T-AOC under basal conditions did not exhibit significant differences among the groups (Figure 5).

### Impact on antioxidant capacity immediately after intense interval training

**FIGURE 6:**
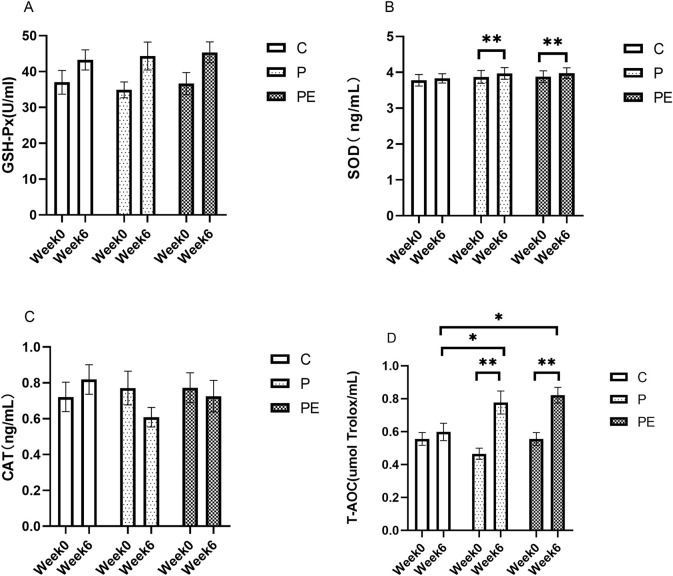
The impact of probiotics supplementation alone or in combination with exercise on the anti-oxidant capacity immediately after high-intensity interval exercise in healthy college students. Results are reported as the mean ± SEM. C, control group (n = 10); P: probiotic group (n = 10); PE: probiotic and aerobic exercise group (n = 10). **(A)** GSH-Px. **(B)** SOD. **(C)** CAT. **(D)** T-AOC. *:*P* < 0.05; **: *P* < 0.01.

**FIGURE 7:**
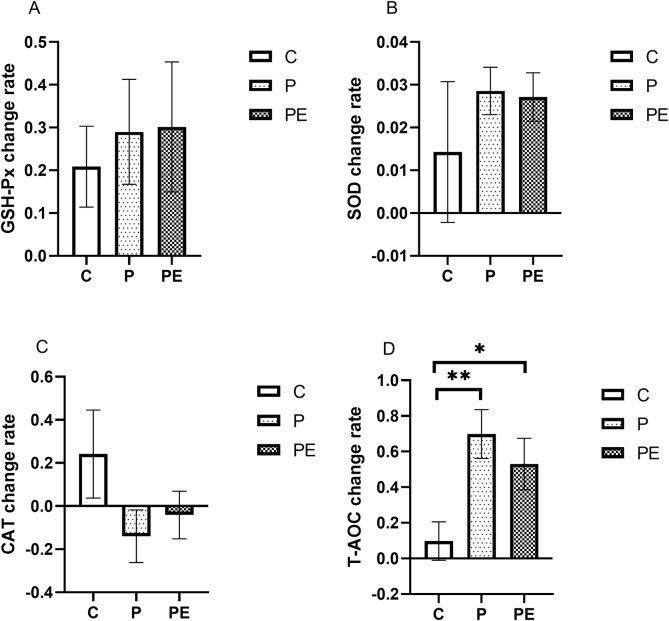
The impact of probiotics supplementation alone or in combination with aerobic exercise on the rate of change of antioxidant capacity immediately after high-intensity interval exercise of college students. Results are reported as the mean ± SEM. C, control group (n = 10); P: probiotic group (n = 10); PE: probiotic and exercise group (n = 10). **(A)** GSH-Px change rate. **(B)** SOD change rate. **(C)** CAT change rate. **(D)** T-AOC change rate. *: *P* < 0.05; **: *P* < 0.01.

This study assessed the effects of 6 weeks of probiotic supplementation, with or without combined aerobic training, on the plasma antioxidant enzymes immediately after high-intensity interval exercise. As shown in Figure 6B, compared to pre-intervention, the SOD activity was significantly increased in both the P group and PE group immediately after high-intensity interval exercise (*P* < 0.05). The P group rose from 3.87 ± 0.18 ng/mL to 3.97 ± 0.16 ng/mL, and the PE group climbed from 3.88 ± 0.16 ng/mL to 3.98 ± 0.15 ng/mL. Similarly, as shown in Figure 6D, the T-AOC level in both the P group and PE group was increased immediately after high-intensity interval exercise from 0.47 ± 0.03 umol Trolox/mL to 0.78 ± 0.07 umol Trolox/mL (*P* < 0.05) and 0.56 ± 0.04 to 0.82 ± 0.05 (*P* < 0.05) in each case. Markedly elevated T-AOC levels were observed in the P and PE groups after 6 weeks of intervention, compared to the C group (*P* < 0.05, Figure 6D). Additionally, T-AOC levels showed a significant interaction effect between the group and time (F = 5.0, *P* = 0.019, η^2^ = 0.36). No significant changes were observed in the GSH-Px (Figures 6A, P > 0.05) and CAT activity (Figures 6C, P > 0.05) in any of the groups before and after the intervention. Furthermore, as shown in Figure 7D, the rate of change in T-AOC level in the P group and PE group was significantly higher than that in the C group (*P* < 0.01 and P < 0.05, respectively). No significant differences were noted in the rates of change for other indices.

### Effects on blood rheology

**FIGURE 8:**
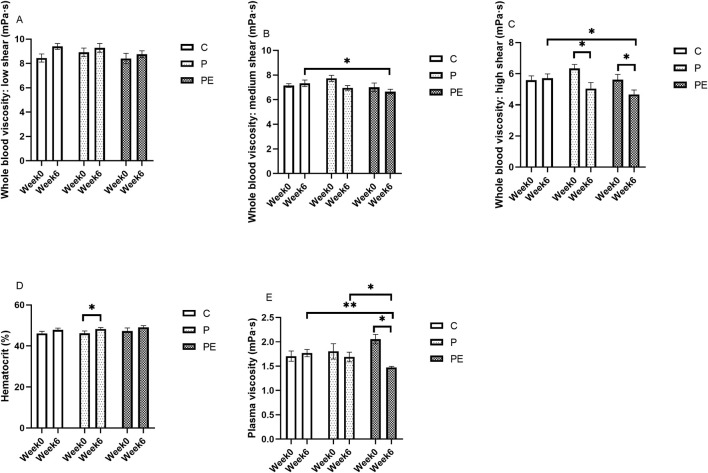
The impact of probiotics supplementation alone or in combination with aerobic exercise on hemorheological parameters at baseline in healthy college students. Results are reported as the mean ± SEM. C, control group (n = 10); P: probiotic group (n = 10); PE: probiotic and aerobic exercise group (n = 10) **(A)** Whole Blood Viscosity: Low Shear. **(B)** Whole Blood Viscosity: Medium Shear. **(C)** Whole Blood Viscosity: High Shear. **(D)** Hematocrit. **(E)** Plasma Viscosity. *:*P* < 0.05; **: *P* < 0.01.

**FIGURE 9:**
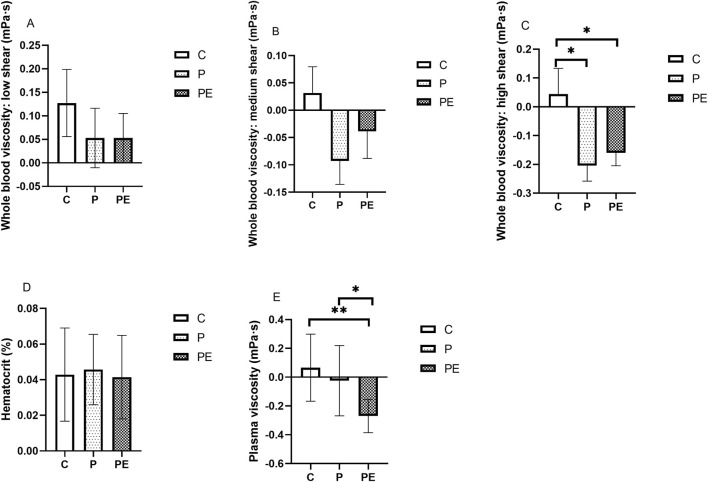
The Influence of Probiotics supplementation alone or in combination with aerobic exercise on the variation rate of hemorheological parameters in healthy university students at baseline. Results are reported as the mean ± SEM. C, control group (n = 10); P: probiotic group (n = 10); PE: probiotic and aerobic exercise group (n = 10). **(A)** Whole Blood Viscosity: Low-Shear Rate Variation. **(B)** Whole blood viscosity: medium shear Rate Variation. **(C)** Whole Blood Viscosity: High-Shear Rate Variation. **(D)** Hematocrit Variation Rate. **(E)** Plasma Viscosity Variation Rate. *:*P* < 0.05; **: *P* < 0.01.

After 6 weeks of probiotic intervention, either alone or in combination with aerobic exercise, its effects on blood rheology were examined before and after the intervention. As shown in Figure 8, at post-intervention, the whole blood viscosity at medium shear in the PE group was significantly lower than in the C group (*P* < 0.05). Compared to baseline, a significant reduction in whole blood viscosity at high shear rates was observed in both the P group and PE group (*P* < 0.05, Figure 8C) from 6.34 ± 0.26 to 5.05 ± 0.39 mPa s at 150 s^-1^, and decreased by 16.76%, respectively. Additionally, the PE group demonstrated a notable decrease relative to the C group in whole blood viscosity at high shear rates after the intervention (*P* < 0.05). Regarding hematocrit, the P group had a significant increase (*P* < 0.05, Figure 8D), while no notable variations were detected in the C and PE groups. Plasma viscosity in the PE group dropped by 28.64% after the intervention, from 2.06 ± 0.10 to 1.47 ± 0.02 mPa s at 150 s^-1^ (*P* < 0.05, Figure 8E). Moreover, the plasma viscosity of the PE group was significantly lower than that of both the C and P groups following the intervention, with a more pronounced difference compared to the C group (Figure 8E). There were no significant differences in whole blood viscosity at low shear rates among the groups (*P* > 0.05, Figure 8A). Furthermore, when compared to the C and P groups, significant differences were detected in the PE group in the rates of change of whole blood viscosity at high shear rates and plasma viscosity (*P* < 0.05, Figures 9C, E). In terms of blood viscosity, results showed a significant interaction effect (F = 5.89, P = 0.01, η2 = 0.40) and time effect (F = 7.04, P = 0.02, η2 = 0.28). No remarkable changes were detected in the rates of change for other indices.

### Effects on aerobic capacity

**FIGURE 10:**
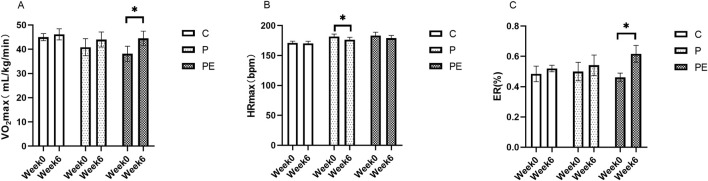
The influence of probiotics supplementation alone or in combination with aerobic exercise on the aerobic capacity of college students. Results are reported as the mean ± SEM. C, control group (n = 10); P: probiotic group (n = 10); PE: probiotic and aerobic exercise group (n = 10). **(A)** VO_2_max. **(B)** HRmax. **(C)** ER. *: *P* < 0.05; **: *P* < 0.01.

**FIGURE 11:**
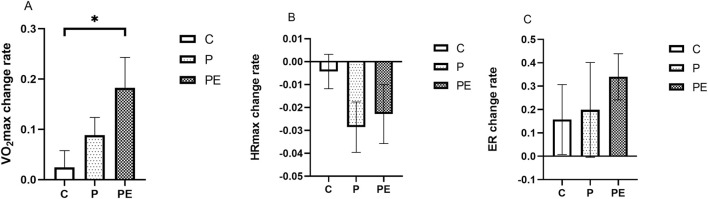
The influence of probiotics supplementation alone or in combination with aerobic exercise on the change rate of aerobic capacity in healthy university students. Results are reported as the mean ± SEM. C, control group (n = 10); P: probiotic group (n = 10); PE: probiotic and aerobic exercise group (n = 10). **(A)** VO_2_max variation rate. **(B)** HRmax change rate. **(C)** ER Variation Rate. *:*P* < 0.05; **: *P* < 0.01.

An assessment was made of the effect of 6 weeks of probiotic supplementation, either alone or combined with aerobic exercise, on participants’ aerobic capacity. As shown in Figure 10, compared to pre-intervention, the VO_2_max demonstrated a marked rise in the PE group, but not in the P group, rising from 38.14 ± 3.11 to 44.5 ± 2.94 mL/kg/min (*P* < 0.01). Meanwhile, the PE group showed a notably greater increase in VO_2_max compared to the C group (*P* < 0.05, Figure 11). The VO_2_max of the C group remained unchanged before and after the intervention. A significant decrease in HRmax was observed in the P group during HIIE (*P* < 0.05, Figure 10B), while the C and PE groups showed no significant changes. Compared to pre-intervention, ER was markedly improved in the PE group, increasing from 0.46% ± 0.03% to 0.62% ± 0.06% (*P* < 0.05, Figure 10C). However, no notable alteration in ER was found in the C or P groups. Additionally, no statistically significant variations were observed in the rates of change for HRmax and ER between the groups (Figures 11B, C).

## Discussion

In summary, our results suggest that probiotic supplementation combined with aerobic exercise is more effective than probiotic supplementation alone in enhancing the antioxidant capacity of the body, but both have the same role in ameliorating the oxidative stress caused by HIIE. These results emphasize the potential of combined strategies to enhance antioxidant capacity, while also demonstrating the notable efficacy of standalone probiotic supplementation in managing oxidative stress after vigorous exercise. Furthermore, increases in VO_2_max and ER following HIIE were observed only in the group that combined probiotics with aerobic exercise, not in the probiotic-only group. This research offers novel scientific insights into the role of probiotics in exercise nutrition and recovery, recommending the integration of probiotics with aerobic exercise in daily training regimens to optimize post-exercise recovery.

Strenuous exercise causes ROS accumulation and instigates oxidative stress in skeletal muscle, which plays a critical role in muscle damage and performance degradation (García-Giménez et al., 2024). Oxidative stress is an adverse physiological response, and the antioxidant defense system in the blood is activated to maintain and restore the dynamic balance between oxidative stress and antioxidant responses. The antioxidant system consists of an enzymatic defense system containing GSH-Px, SOD, and CAT, and a nonenzymatic antioxidant system (Zhao et al., 2023). 8-OHdG is the most common biomarker of DNA damage in urine and blood samples and is recognized as a biomarker of oxidative DNA damage (Spoto et al., 2025). This study revealed that SOD activity and T-AOC levels exhibited a marked elevation in the P group immediately after HIIE compared to pre-intervention. Moreover, post-intervention, the level of 8-OHDG and its rate of change immediately after HIIE were notably diminished in the P group compared to the C group, suggesting that probiotics supplementation may help reduce oxidative damage caused by exercise. Published investigations support our findings. Kola et al. (Kayacan et al., 2022) found that probiotic supplementation by itself had no effect on oxidative stress levels in the basal state of rats. However, when combined with chronic exercise, probiotics signif-icantly reduced the oxidative damage caused by the exercise. Two weeks of probiotic yogurt supplementation in healthy subjects significantly increased the levels of GSH-Px, SOD, and T-AOC, and decreased the MDA level after sub-exhaustive exercise (Mazani et al., 2018). The most recent study also found that supplementation with probiotics (including *Bacillus longum* CECT7347, *Lactobacillus casei* CECT9104, and *Lactobacillus rhamnosus* CECT8361) significantly reduced the levels of serum MDA and urinary 8-OHDG in healthy men after high-intensity cycling (Sánchez Macarro et al., 2021). However, in our study, there were no changes in basal state GSH-Px and T-AOC activities after probiotic supplementation, which is inconsistent with the results of previous studies (Mazani et al., 2018), possibly because the probiotic species and subjects were different. It is important to note that our results reflect the acute response to a single bout of high-intensity exercise, which tends to increase oxidative stress in the short term. However, previous studies have shown that long-term HIIE can reduce oxidative stress through adaptation mechanisms, including the upregulation of antioxidant enzymes such as SOD and CAT (Sarvasti et al., 2020; Guo et al., 2025). In addition, studies have shown that long-term HIIE combined with supplements such as green tea or caffeine can have beneficial effects in mitigating oxidative stress. (Ghasemi et al., 2020; Alkhatib et al., 2020). Nevertheless, whether long-term HIIE combined with probiotic supplementation can similarly improve systemic antioxidant capacity remains unclear and warrants further investigation.

Our data showed a significant increase in basal GSH-Px activity in the PE group compared to pre-intervention levels. No significant changes were observed in the P group. It is suggested that probiotics combined with exercise are more effective in enhancing basal antioxidant capacity. Aerobic exercise has been shown to increase T-AOC levels, enhance the activities of CAT, GSH-Px, and SOD (Zarrindast et al., 2021), and decrease MDA level (Sun et al., 2023). However, given the lack of a separate aerobic exercise group in this study, it is not possible to determine whether the increase in basal state GSH-Px activity was a result of aerobic exercise or a synergistic effect of probiotics and aerobic exercise. Notably, no significant difference was observed between the P and PE groups in terms of improving oxidative stress caused by HIIE in this study. Suggesting that probiotics may help regulate exercise-induced oxidative stress by enhancing SOD and T-AOC, while reducing 8-OHdG during the acute recovery phase following HII. Additionally, in terms of T-AOC levels, the results showed a significant interaction effect between the group and time. Post-hoc comparisons revealed that the combination of probiotics and aerobic exercise led to a more significant increase in T-AOC levels than probiotic supplementation alone, particularly at the post-intervention time point. The effectiveness of probiotics suggests that they can be used as a convenient and effective stand-alone strategy, particularly for those who do not regularly participate in aerobic exercise but can mitigate oxidative stress through probiotic supplementation. Given its convenience and accessibility, probiotic supplementation may be particularly valuable for college students and younger individuals who lack regular aerobic training.

This study found that after the intervention, the high shear rate blood viscosity in the P group significantly decreased, indicating that probiotic supplementation may reduce blood viscosity. This outcome aligns with previous research, which suggests that probiotic supplementation can regulate vascular endothelial function and inflammatory responses (Hofeld et al., 2021; Zhai et al., 2022). Probiotics likely achieve this by modulating the gut microbiota, which reduces systemic inflammation and improves endothelial function. This decrease in inflammation can enhance vascular health and lower blood viscosity (Liu et al., 2025). Additionally, the reduction in blood viscosity in the PE group may be partly due to aerobic exercise, which improves endothelial function and promotes vasodilation, resulting in smoother blood flow. Aerobic exercise boosts nitric oxide production, relaxing blood vessels, improving circulation, and lowering blood viscosity (Shing et al., 2024; Meier et al., 2023). This enhanced endothelial function helps regulate blood flow more efficiently. Furthermore, after the intervention, both the high shear rate whole blood viscosity and plasma viscosity were significantly decreased in the PE group. The levels of high shear rate whole blood viscosity and plasma viscosity were significantly lower in the PE group than in the C group, and plasma viscosity was significantly lower than in the P group. These results suggest that the combination of probiotics and aerobic exercise may be more effective in improving blood rheology than probiotics supplementation alone. Thus, the improvement in blood rheology may be partly attributed to the effects of aerobic exercise. The synergy between probiotics and aerobic exercise likely enhances the beneficial effects on blood viscosity and circulation, as probiotics reduce systemic inflammation while aerobic exercise improves vascular function and blood flow. Similarly, aerobic training has been shown to significantly improve cardiovascular function and oxygen uptake capacity (Collins et al., 2024; Mardyła et al., 2023). Regular interval aerobic training can significantly improve blood rheological parameters (Mougin et al., 2024). Mardyła et al. found that regular training can improve blood rheological functions, such as reducing fibrinogen concentration, plasma viscosity, and aggregation index in the rowers (Mardyła et al., 2023). Additionally, 30 minutes of aerobic exercise three times a week also can improve hematocrit and plasma viscosity (Marchewka et al., 2015). Considering the changes in blood viscosity at high shear rates and viscosity of blood plasma, smoother blood flow in the blood vessels is facilitated, improving circulation, enhancing the fluidity of blood, and increasing the capacity for oxygen and nutrient transport. The improvement of blood rheology in the PE group may contribute to the improvement of aerobic capacity to some extent. This study also found that the VO_2_max was significantly higher in the PE group compared to pre-intervention, which may be related to the improvement of blood rheology.

VO_2_max is one of the key indicators of an individual’s aerobic capacity, reflecting the ability of the human body to inhale, transport, and utilize oxygen (Zhang et al., 2023). Evidence from earlier studies indicates that probiotic supplementation may boost the VO_2_max of participants. A study involving 66 long-distance runners found that a 12-week probiotic supplementation led to an increase in VO_2_max in both male and female participants (Smarkusz-Zarzecka et al., 2020). Additionally, VO_2_max was also significantly elevated in probiotic-supplemented male soccer players (Imanian et al., 2024) and badminton players (Salleh et al., 2021). The improvement of aerobic capacity by probiotics supplementation may be because probiotics can balance gut microbiota (Zhang et al., 2024), thereby promoting iron absorption and hemoglobin synthesis, and short-chain fatty acids, metabolites produced by the gut microbiota, directly enhance hemoglobin synthesis (Takahashi et al., 1975; Li et al., 2023). However, the subjects of the above studies were athletes, and the effect of exercise on VO_2_max was not excluded. Conversely, some studies have shown no change in VO_2_max after probiotic supplementation (Michalickova et al., 2016), possibly due to differences in probiotic formulations, dosages, duration of intervention, and experimental designs. The study results showed an upward trend in VO_2_max in the P group after the intervention period, though no substantial change was found. In contrast, the VO_2_max increased significantly in the PE group, and the change in VO_2_max was significantly more pronounced in the PE group compared to the C group. It is suggested that the results may be due to the positive effect of aerobic exercise. Additionally, this study examined the HRmax levels during high-intensity interval training. A notable decrease in HRmax was found in the P group, which is consistent with the findings of Paulina et al. (Mazur-Kurach et al., 2022) who found that probiotic supplementa-tion could significantly reduce the HRmax in road cyclists. However, the PE group showed no significant difference. Thus, further studies should explore the optimization of combined probiotic and aerobic exercise interventions. Furthermore, this study found a significant increase in ER after probiotics combined with aerobic training intervention, whereas no notable change was observed in the group receiving probiotic supplementation alone. Suggesting that the improvement in ER may be caused by aerobic exercise, this result supports the key role of exercise training in regulating ER (Fukuba et al., 1999; Rahimi and Beaven, 2024). The enhancement in ER may contribute to post-exercise recovery and also reflect the body’s aerobic capacity. In summary, probiotics combined with aerobic exercise are more helpful for aerobic capacity than supplementing with probiotics alone.

The probiotics investigated in this study have been shown to modulate gut microbiome composition. Supplementation with *Lactobacillus zhang* significantly altered the gut microbiota composition of healthy volunteers (Zhang et al., 2014), increasing the abundance of beneficial bacteria (*Lactobacillus* and *Bifidobacterium*), short-chain fatty acids-producing bacteria (such as *Prevotella*), and anti-inflammatory bacteria (*Faecalibacterium*), while significantly reducing the number of opportunistic pathogens (*Clostridium* and *Enterococcus faecalis*). Similarly, *Lactobacillus plantarum P-8* (Wang et al., 2014) also increased the abundance of *Bifidobacterium* in the gut of healthy volunteers and decreased the abundance of *Clostridium.* Unfortunately, gut microbiota was not measured in the study, which should be taken into account in future studies. In addition, factors limiting this study included a relatively small sample size and the lack of a separate aerobic exercise group. Future studies should explore the long-term influence of probiotics and aerobic recovery processes on oxidative stress and offer evidence-based recommendations for athletes and young individuals.

## Conclusion

In conclusion, combining probiotics with aerobic exercise not only enhances antioxidant capacity but also improves aerobic performance, making it a promising strategy for optimizing exercise recovery and performance in college students.

## Data Availability

The raw data supporting the conclusions of this article will be made available by the authors, without undue reservation.
